# Perinatal Treatment with Leptin, but Not Celastrol, Protects from Metabolically Obese, Normal-Weight Phenotype in Rats

**DOI:** 10.3390/nu14112277

**Published:** 2022-05-29

**Authors:** Bàrbara Reynés, Margalida Cifre, Andreu Palou, Paula Oliver

**Affiliations:** 1 Nutrigenomics, Biomarkers and Risk Evaluation Group, University of the Balearic Islands, 07122 Palma, Spain; barbara.reynes@uib.es (B.R.); marga.cifrec@gmail.com (M.C.); paula.oliver@uib.es (P.O.); 2 Health Research Institute of the Balearic Islands (IdISBa), 07010 Palma, Spain; 3 CIBER of Pathophysiology of Obesity and Nutrition (CIBEROBN), Instituto de Salud Carlos III, 28029 Madrid, Spain

**Keywords:** leptin, celastrol, perinatal nutrition, metabolically obese, normal weight

## Abstract

Perinatal nutrition has a well-known influence on obesity susceptibility. We previously demonstrated the protective anti-obesity effects of perinatal leptin administration. Celastrol is a natural compound acting as a leptin sensitizer with anti-obesity effects when administered in adult animals. Here, we aimed to determine if perinatal treatment with leptin, celastrol, or their combination was able to improve metabolic health in animals fed an isocaloric high-fat (HF) diet. Leptin and/or celastrol or their vehicle were administered orally to rats during the suckling period. After weaning, animals were chronically pair-fed with an HF diet provided isocaloric to the intake of a normal-fat diet by control animals to avoid obesity. Isocaloric HF feeding in vehicle-treated animals resulted in metabolic features characteristic of the metabolically obese, normal-weight (MONW) phenotype, i.e., obesity-related disturbances without increased body weight. Leptin treatment prevented liver fat deposition and insulin resistance, induced greater insulin and leptin signaling capacity, decreased gene expression of orexigenic signals at the hypothalamic level, and induced browning in retroperitoneal adipose tissue. However, celastrol treatment did not provide any protective effect and resulted in greater size of the retroperitoneal adipose depot, higher circulating glucose and insulin levels, and decreased leptin sensitivity capacity in adipose tissue. The co-administration of leptin ameliorated the negative effects of celastrol on the retroperitoneal depot, inducing browning and decreasing its size. In conclusion, the perinatal administration of leptin, but not celastrol, provided protection against the consequences of dietary unbalances leading to an MONW phenotype in adulthood.

## 1. Introduction

One’s early life environment, including perinatal nutrition, has an exceptional influence on the metabolic phenotype in adulthood [1,2]. In this sense, different studies have revealed the beneficial effects of breastfeeding versus formula feeding in terms of obesity prevention [3,4], which could be related to the different hormones and compounds present in breast milk [5,6]. One of these molecules, with exceptional activity in perinatal programming, is leptin. This hormone, mainly produced by adipose tissue but present in maternal milk, acts as an essential nutrient during lactation for the programming of a lean phenotype, and we have demonstrated that leptin treatment during the suckling period protects against diet-induced obesity in adulthood [7,8]. For instance, using rodents, our group revealed that perinatal leptin treatment improves, among other parameters, insulin and leptin sensitivity in adulthood and increases the oxidative capacity, which results in better handling of excess fuel [7,8,9]. In addition, leptin is also a key hormone acting in the prenatal period, being involved in the neuronal development in the fetus, which is crucial for the proper development of the homeostatic system for body weight control [10].

Beyond the aforementioned effects of leptin on perinatal programming, this hormone carries information from peripheral energy stores and is involved in the maintenance of energy homeostasis, increasing energy expenditure [11]. However, in the obese state, despite the presence of elevated leptin levels, this is ineffective in increasing energy expenditure and decreasing body weight due to leptin insensitivity or leptin resistance [12]. In this sense, celastrol, a pentacyclic triterpene extracted from the roots of *Tripterygium wilfordi*, appeared as an interesting anti-obesity compound due to its powerful role as a leptin sensitizer [13]. The oral administration of celastrol to hyperleptinemic-diet-induced obese rodents has been shown to cause food intake reduction, accompanied by a decreased body weight and decreased visceral fat accumulation, improving metabolic alterations related to the intake of the high-fat (HF) diet [13,14,15]. Given the powerful short-term effect of celastrol on leptin action, this new bioactive compound appears as an interesting candidate to modulate long-term leptin action by acting on metabolic programming with the aim of preventing obesity.

The prevalence of obesity is closely related to adherence to an unhealthy lifestyle, mainly associated with an excessive intake of inadequate diets and sedentary habits. However, the intake of unbalanced diets (rich in fats or simple carbohydrates) is not necessarily related to the development of obesity. For instance, the intake of diets with an unbalanced macronutrient proportion in a controlled caloric intake is linked to the metabolically obese, normal-weight (MONW) phenotype [16,17,18,19]. In spite of having a thin appearance, individuals with MONW display several metabolic features associated with an obese state, such as higher adiposity and increased ectopic fat accumulation, increased hyperinsulinemia, and insulin resistance, among others [20,21,22]. Particularly, MONW affects around 20% of the worldwide population [23]. This constitutes a serious public health concern since an important part of the population could be at a higher metabolic risk that could not be diagnosed due to a lack of obesity. Hence, there is interest in perinatal interventions aimed at conferring protection and preventing the appearance of the MONW phenotype.

Therefore, in this study, we aimed to evaluate the metabolic effects of celastrol administered during lactation alone or in combination with leptin on adult animals chronically fed an HF diet administered isocalorically to a control diet, which is known to induce the MONW phenotype.

## 2. Materials and Methods

### 2.1. Animal and Experimental Design

Animal procedure followed in this study was approved by the Bioethical Committee of the University of the Balearic Islands and followed the guidelines for the use and care of laboratory animals of the university. Two-month-old virgin female Wistar rats were mated with male rats (Charles River Laboratories; Barcelona, Spain). After matching, females were placed in individual cages with free access to water and food. On day 1 after delivery, excess pups in each litter were removed to keep 10 pups per dam. Male pups from a total of 8 litters were randomly assigned into 4 groups receiving different experimental treatments from day 1 to day 20 of lactation during the first 2 h of the beginning of the light cycle: Control (n = 16), leptin (n = 10), celastrol (n = 11) and a group treated with leptin and celastrol, Lep+Cel (n = 8). All treatments were administered orally. A solution of recombinant murine leptin (PeproTech, London, UK) and celastrol (BOC Sciences; Shirley, NY, USA) dissolved in Captisol^®^ was administered to leptin and celastrol-treated groups, respectively. The amount of leptin administered to the animals was calculated as five times the average amount of daily leptin intake from breast milk, calculated in a previous study by our group [24]. Celastrol-treated pups received 10 mg celastrol/kg of body weight, the same amount that was reported by other authors to protect against obesity when administered to adult mice [13], which have similar body weight to our neonate rats. The Lep+Cel group received the sum of both treatments (administered mixed in a single shot) in order to analyze a possible synergistic effect of the two compounds. Finally, the Control group received the same volume of the vehicle, Captisol^®^ (Ligand Pharmaceuticals, San Diego, CA, USA), as the maximum volume administered among the treatments to the different experimental groups. Captisol^®^ is a modified cyclodextrin, which was used as a vehicle because its chemical structure improves solubility, stability, and bioavailability of administered compounds [25]. During the suckling period, rats were weighed every day before receiving the corresponding treatment. On day 21, after weaning, male rats were single-caged and kept on a normolipidic control balanced diet (D12450B, Research Diets). On day 23, the Control group rats were randomly assigned to two groups: NF-Control (n = 8), fed ad libitum a standard chow diet with 10% calories from fat (D12450B, Research Diets); and HF-Control (n = 8), fed a chow diet with 60% calories from fat (D12492, Research Diets), administered under isocaloric conditions to NF-controls, as previously described [26], to prevent development of obesity. Moreover, treated groups (now named HF-Leptin, HF-Celastrol, and HF-Lep+Cel) received the same hyperlipidic diet as the HF-Control group, also administered under isocaloric conditions relative to the Control group. All diets were purchased from Brogaarden (Gentofte; Denmark). Animals were maintained on these diets until 14 weeks of age. During the whole intervention period, rats were housed under standard conditions, with a controlled temperature (22 °C) and a 12 h light, 12 h dark cycle (light on from 08:00 to 20:00 h).

Body weight was recorded three times a week. Body fat composition (fat mass) was determined at 14 weeks of age using an EchoMRI-700TM (Echo Medical Systems, LLC; Houston, TX, USA). At the end of the experimental process, the animals were sacrificed by decapitation and different white adipose tissue (WAT) depots (epididymal, eWAT; retroperitoneal, rWAT; inguinal, iWAT; and mesenteric, mWAT), interescapular brown adipose tissue (BAT), liver, and hypothalamus were rapidly removed, weighed and frozen in liquid nitrogen and stored at −80 °C until RNA analysis. Troncular blood was collected from the neck, stored at 4 °C for 1–3 h, and then centrifuged at 1000× *g* for 10 min at 4 °C to collect the serum. In addition, at the end of the experimental period, animals were subjected to a 12–14 h nocturnal fasting for serum collection in order to determine HOMA-IR index using the formula of Matthews [27].

### 2.2. Adiposity Index

Adiposity was determined by an adiposity index computed for each rat as the sum of the mass of all WAT depots (epididymal, inguinal, mesenteric, and retroperitoneal) expressed as a percentage of total body weight.

### 2.3. Circulating Parameters (Glucose, Insulin, Leptin)

Blood glucose concentration was measured using Accu-Chek Glucometer (Roche Diagnostics; Barcelona, Spain). Serum insulin and leptin levels were measured using enzim-linked immunoabsorbent assay (ELISA) kits (from Mercodia AB; Upsala, Sweden, and R&D Systems; Minneapolis, MN, USA, respectively).

### 2.4. Homeostatic Model Assessment of Insulin Resistance (HOMA-IR)

HOMA-IR was measured from fasting glucose and insulin levels using the formula of Matthews et al. [27].

### 2.5. Quantification of Liver Lipid Levels

Lipid extracts were obtained from liver and used to calculate total fat content as we previously described [28] and to measure triacylglycerol content using the Serum Triglyceride Determination Kit (Sigma Diagnostics; Madrid, Spain).

### 2.6. Liver Histological Analysis

Liver samples were fixed and stained as we previously described [29]. Hematoxylin-eosin-stained tissue sections were analyzed to detect the presence of steatosis.

### 2.7. Histological and Immunohistochemistry Analysis of UCP1 in Retroperitoneal White Adipose Tissue

The number of adipocytes was counted in hematoxylin-eosin-stained tissue sections of rWAT. Results were expressed as the number of adipocytes per area (mm^2^). For immunohistochemistry analysis, serial sections of rWAT fixed samples were incubated with normal goat serum 2% in PBS pH 7.3 to block unspecific sites, and then with primary rabbit polyclonal UCP1 antibody (GeneTex International Corporation; Irvine, CA, USA) diluted 1:200 in PBS overnight at 4 °C. Sections were then incubated with biotinylated anti-rabbit IgG secondary antibody (Vector Laboratories; Burlingame, CA, USA) diluted at 1:200, and finally with ABC complex (Vectastain ABC kit, Vector; Burlingame, CA, USA). Peroxidase activity was revealed with Sigma Fast 3,3TM-diaminobenzidine (Sigma-Aldrich; Madrid, Spain) as substrate. Sections were counterstained with hematoxylin and mounted in Eukitt (O. Kindler; Freiburg, Germany). Images were acquired with a Zeiss Axioskop 2 microscope equipped with an AxioCam ICc3 digital camera and AxioVision 40V 4.6.3.0 software (Carl Zeiss).

### 2.8. Total RNA Isolation

Total RNA from hypothalamus, epididymal and inguinal WAT, BAT, and liver were isolated using Tripure reagent (Roche Diagnostics; Barcelona, Spain) and purified by precipitation with 3M sodium acetate and absolute ethanol. RNA was quantified using NanoDrop ND-1000 spectrophotometer (NanoDrop Technologies Inc.; Wilmington, DE, USA), and its integrity was confirmed by agarose gel electrophoresis.

### 2.9. Real-Time Reverse Transcriptase Polymerase Chain Reaction (RT-qPCR)

Messenger RNA expression of key genes involved in energy homeostasis was analyzed in liver, different white and brown adipose tissue depots, and in hypothalamus. Reverse transcription was carried out in liver, BAT, and hypothalamus samples using 250 ng of RNA reverse-transcribed to cDNA according to Applied Biosystems’ instructions [29], and in WAT using 50 ng of RNA reverse-transcribed to cDNA according to iScript cDNA synthesis kit instructions (BIO-RAD; Madrid, Spain) [30] in an Applied Biosystems 2720 Thermal Cycler (Applied Biosystems; Madrid, Spain). Each qPCR was performed from diluted (1/20 for liver and 1/10 for the other tissues) cDNA according to Applied Biosystems’ instructions with some modifications [29]. The threshold cycle (Ct) was calculated by instrument’s software (StepOneSoftware v2.2, from Applied Biosystems, Waltham, MA, USA), and the relative expression of each mRNA was calculated as a percentage of NF-Control rats using the 2-ΔCt method [31]. Gene expression data from all analyzed tissues were normalized against the reference gene *Guanosine diphosphate dissociation inhibitor* 1 (*Gdi1*), chosen based on microarray data from our group, which show its stability in control and diet-induced obese animals [32]. Primers for the different genes analyzed (Sigma-Aldrich Química SA; Madrid, Spain) are described in Supplementary Table S1.

### 2.10. Statistical Analysis

All data are expressed as the mean ± SEM. The dietary effects were analyzed using U Mann–Whitney test, and differences between treatments were analyzed using one-way ANOVA or U Mann–Whitney test. LSD post hoc test was used after ANOVA analysis. The parameters that did not meet the required rules for one-way ANOVA were transformed to Ln or Log10. Threshold of significance was defined at *p* < 0.05 and is indicated when different. Analyses were performed with SPSS for Windows (SPSS; Chicago, IL, USA).

## 3. Results

### 3.1. Body Weight, Adiposity, and Circulating Parameters

**Figure 1:**
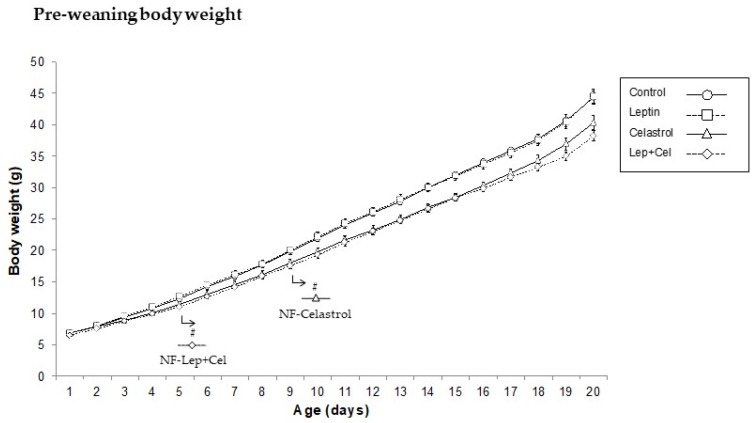
Body weight evolution during lactation in pups treated with vehicle (Control), leptin, celastrol, and with leptin and celastrol (Lep+Cel). Data represent means ± SEMs (n = 8–16). Statistics: The # symbol shows the significance vs. Control animals (U Mann–Whitney, *p* < 0.05).

During the pre-weaning suckling period, and consistent with the previously reported anti-obesity effect of celastrol, animals from both celastrol and Lep+Cel groups showed a decrease in body weight compared to control animals, while treatment with leptin did not affect weight gain (Figure 1). As expected, pair-feeding with an HF diet for 11 weeks after weaning did not affect body weight but increased fat mass content in HF-fed rats (Table 1). Thus, at the end of the intervention, all the HF-fed groups presented an increased fat mass content and increased adiposity index. However, differences were evident depending on the perinatal treatment received. In contrast to the other HF-fed groups, leptin-treated animals did not show a greater size of the epididymal WAT, in accordance with the expected antilipogenic role described for this hormone [7]. However, perinatal treatment with celastrol resulted in a greater size of the retroperitoneal WAT in comparison to both NF and HF-Controls (U Mann–Whitney, *p* < 0.05). In fact, in a one-way ANOVA comparison, animals in the HF-Celastrol group had a greater fat mass (measured using an EchoMRI analyzer) compared to NF- and HF-Controls. Nevertheless, when celastrol was administered in combination with leptin, the greater size of the rWAT depot was not observed in the HF-Lep+Cel group. In fact, this group presented intermediate fat mass levels between those of the HF-Celastrol and HF-Leptin groups (one-way ANOVA, *p* < 0.05). In addition, the HF-Lep+Cel group was the only one that presented a greater size of the BAT (U Mann–Whitney, *p* < 0.05).

As expected, circulating parameters related to insulin resistance (e.g., fasting circulating glucose and HOMA index), as well as circulating leptin levels, were increased as a result of HF diet feeding (U Mann–Whitney, *p* < 0.05) (Table 1). The alteration of these adiposity-related parameters in the absence of obesity is characteristic of the MOWN phenotype. However, leptin-treated animals did not present alterations indicative of insulin resistance despite the chronic HF diet intake, and, in addition, they presented lower circulating leptin levels compared to the HF-Control group, all this being indicative of a healthier metabolic status. In contrast, no beneficial effects were observed for these parameters related to insulin resistance and adiposity as a result of perinatal treatment with celastrol, although animals co-treated with leptin had normal serum insulin levels (U Mann–Whitney test) and lower leptin levels than animals treated with celastrol alone (one-way ANOVA, *p* < 0.05).

### 3.2. Liver Fat Deposition and Histological Analysis

**Figure 2:**
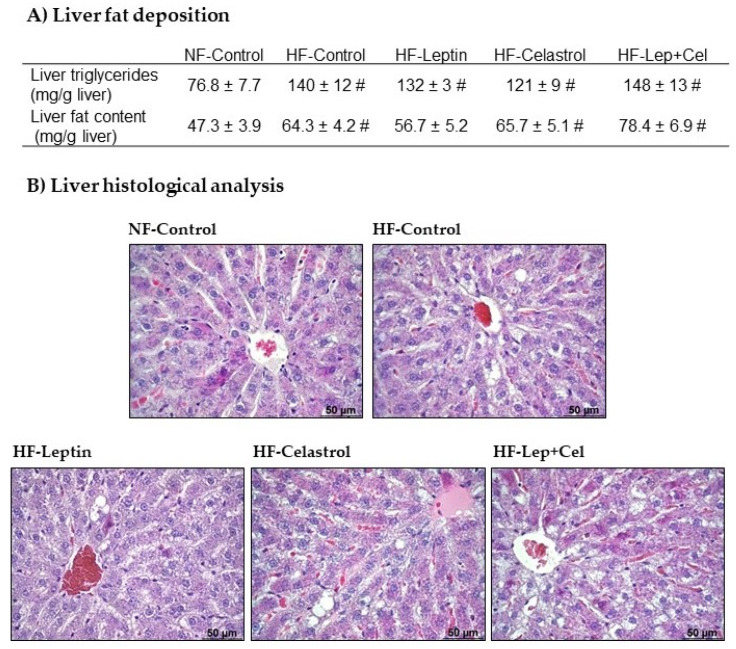
Liver analysis. Liver fat content (**A**) (triglycerides and total lipids) and (**B**) liver sections stained with hematoxylin and eosin in adult control rats fed a normal-fat diet (NF-Control) and in adult rats that were treated during lactation with vehicle (Control), leptin, celastrol, or leptin and celastrol (Lep+Cel), and subsequently pair-fed for 11 weeks with high-fat diet (HF) administered under isocaloric conditions to the NF-Control group. In (**A**), data represent means ± SEMs (n = 8–10). Statistics: The # symbol shows the significance of all the HF-groups vs. the NF-Control group (U Mann–Whitney, *p* < 0.05). In (**B**), representative sections are shown.

Total fat liver content was increased by HF pair-feeding, except in leptin-treated animals, whereas liver triglycerides increased with hyperlipidic diet feeding, regardless of perinatal treatment (U Mann–Whitney, *p* < 0.05) (Figure 2A). Microscopic observation of liver sections confirmed the presence of microvesicular steatosis in the liver of the HF-fed groups; smaller lipid vacuoles could be visually observed in the HF-Leptin group (Figure 2B), consistent with the lower total fat content. No evidence of fibrosis was evident in any of the groups.

### 3.3. Liver Gene Expression Analysis

**Figure 3:**
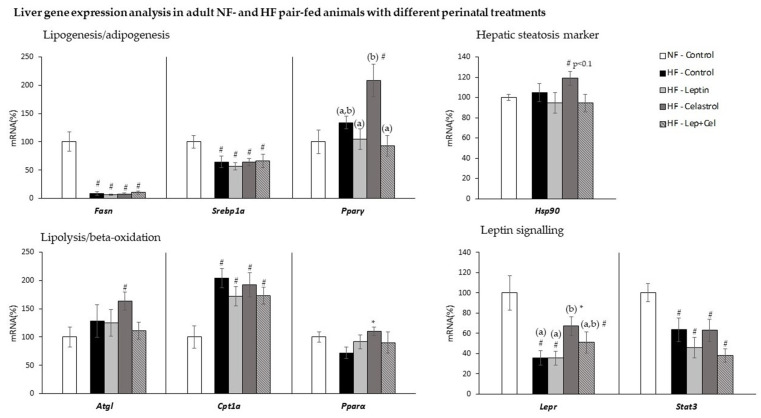
Liver gene expression analysis in adult control rats fed a normal-fat diet (NF-Control) and in adult rats which were treated during lactation with vehicle (Control), leptin, celastrol, or leptin and celastrol (Lep+Cel) and subsequently pair-fed for 11 weeks with high-fat diet (HF) administered under isocaloric conditions to the NF-Control group. mRNA expression was measured by RT-qPCR. Data represent means ± SEMs (n = 8–10) of ratios of specific mRNA levels relative to *Gdi* (used as reference gene), expressed as a percentage of the value of the NF-Control group that was set to 100%. Statistics: One-way ANOVA was only performed in the HF-fed animals. Data not sharing a common letter (a, b) are significantly different. LSD post hoc was used after ANOVA analysis. The # symbol shows the significance of all the HF-fed groups vs. the NF-Control group (U Mann–Whitney, *p* < 0.05); the * symbol shows the significance of the HF-treated groups vs. the HF-Control group (U Mann–Whitney, *p* < 0.05).

As shown in Figure 3, as an adaptation to higher fat intake, HF pair-feeding decreased liver gene expression of the lipogenic genes *Fasn* and *Srebp1a* and increased the expression of the fatty acid beta-oxidation gene *Cpt1a*, regardless of the treatment received during the suckling period (U Mann–Whitney *p* < 0.05). In addition, in celastrol-treated animals, a greater mRNA expression was observed for the lipolytic *Atgl* and for the adipogenic *Pparγ* genes; these changes were not evident for the HF-Lep+Cel group. Moreover, hyperlipidic diet feeding decreased the expression of leptin-signaling-related genes (*Lepr* and *Stat3*) in the liver, except in those animals treated with celastrol administered alone that presented increased *Lepr* expression compared to the HF-Control group (U Mann–Whitney and one-way ANOVA, *p* < 0.05). Finally, a trend towards a greater mRNA expression of *Hsp90*, evaluated as a marker of steatosis, was only observed in the HF-Cel group compared to the other HF-fed animals (U Mann–Whitney, *p* < 0.1).

### 3.4. White Adipose Tissue Gene Expression and Histological and Immunohistochemical Analysis

**Figure 4:**
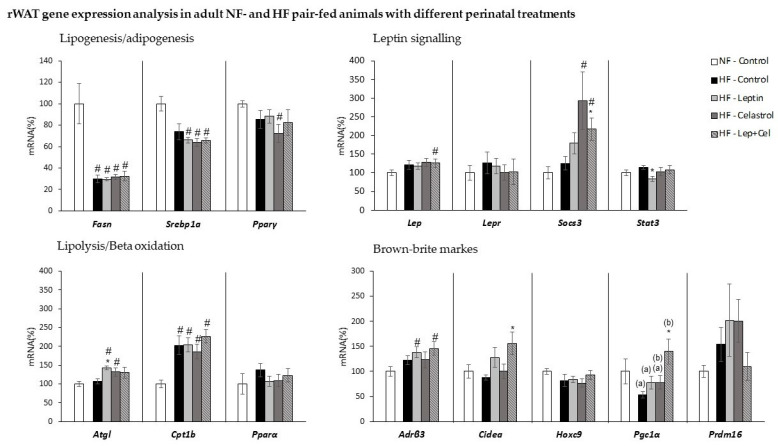
Retroperitoneal WAT (rWAT) gene expression analysis in adult control rats fed a normal-fat diet (NF-Control) and in adult rats that were treated during lactation with vehicle (Control), leptin, celastrol, or leptin and celastrol (Lep+Cel) and subsequently pair-fed for 11 weeks with high-fat diet (HF) administered under isocaloric conditions to the NF-Control group. mRNA expression was measured by RT-qPCR. Data represent means ± SEMs (n= 8–10) of ratios of specific mRNA levels relative to *Gdi* (used reference gene), expressed as a percentage of the value of the NF-Control group that was set to 100%. Statistics: One-way ANOVA was performed only in the HF-fed animals. Data not sharing a common letter (a, b) are significantly different. LSD post hoc was used after ANOVA analysis. The # symbol shows the significance of all the HF-fed groups vs. the NF-Control group (U Mann–Whitney, *p* < 0.05); the * symbol shows the significance of the HF-treated groups vs. the HF-Control group (U Mann–Whitney, *p* < 0.05).

**Figure 5:**
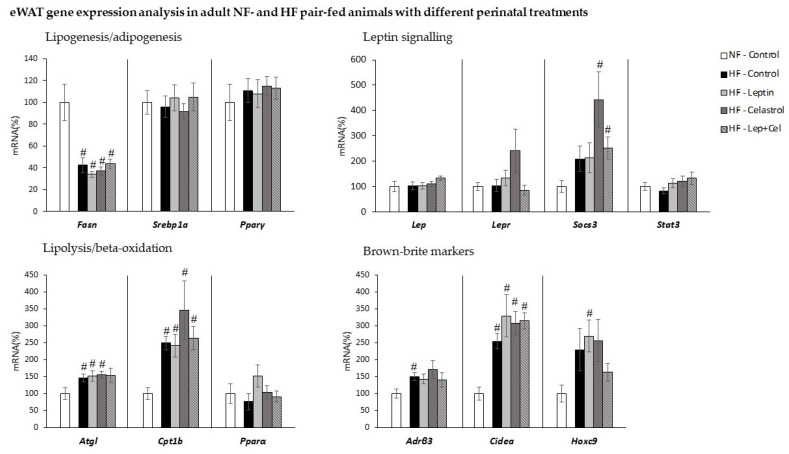
Epididymal WAT (eWAT) gene expression analysis in adult control rats fed a normal-fat diet (NF-Control) and in adult rats that were treated during lactation with vehicle (Control), leptin, celastrol, or leptin and celastrol (Lep+Cel) and subsequently pair-fed for 11 weeks with high-fat diet (HF) administered under isocaloric conditions to the NF-Control group. mRNA expression was measured by RT-qPCR. Data represent means ± SEMs (n= 8–10) of ratios of specific mRNA levels relative to *Gdi* (used reference gene), expressed as a percentage of the value of the NF-Control group that was set to 100%. Statistics: The # symbol shows the significance of all the HF-fed groups vs. the NF-Control group (U Mann–Whitney, *p* < 0.05).

Figure 4 and Figure 5 show gene expression analysis in retroperitoneal and epididymal WAT, respectively, selected as representative depots. The rWAT was selected based on our previous observation, pointing it as a highly prone WAT to develop an energy-dissipative browning profile in response to an HF dietary stimulus, while eWAT has a behavior more typical of a WAT tissue involved in lipid storage [33]. Gene expression regulatory patterns of lipogenic/adipogenic and lipolytic/fatty acid-oxidation-related genes were similar for both adipose depots and similar to that observed in the liver. That is, isocaloric intake of a fat-rich diet induced an adaptive response that was evidenced as a decreased expression in both tissues of the key lipogenic *Fasn* gene in all the HF-groups, regardless of treatment received during suckling, and decreased expression of *Srebp1a* in the HF-Leptin, HF-Cel, and HF-Cel+Lep groups only in the rWAT (U Mann–Whitney, *p* < 0.05). In contrast, HF feeding induced greater expression of key genes involved in beta-oxidation and lipolysis: *Cpt1β* mRNA levels were greater in all the HF-fed groups, and greater *Atgl* mRNA expression was also observed in different HF groups (U Mann–Whitney, *p* < 0.05).

**Figure 6:**
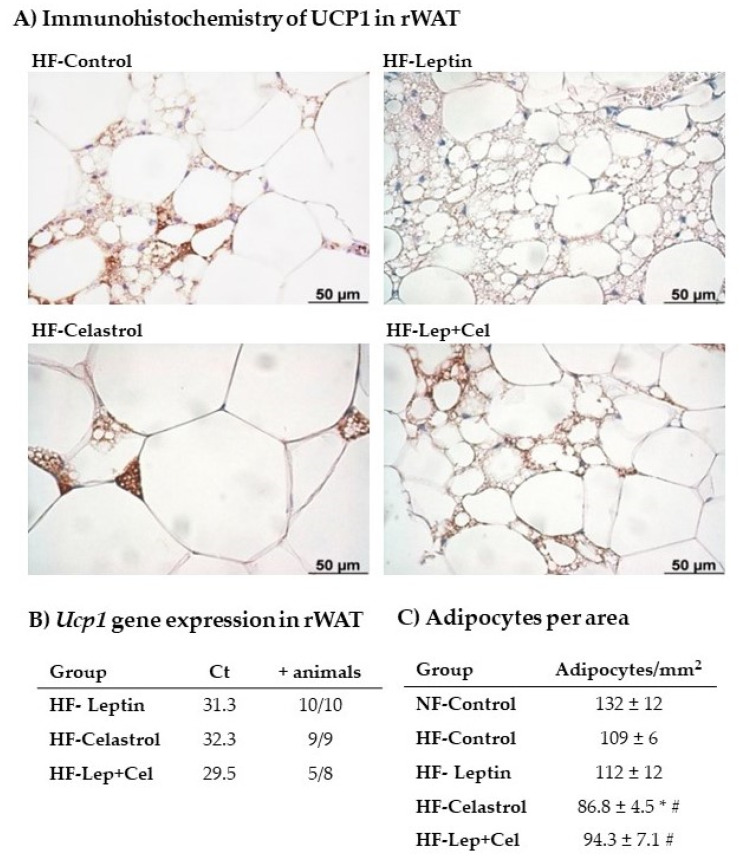
Retroperitoneal WAT (rWAT) analysis. (**A**) UCP1 immunostaining; (**B**) *Ucp1* gene expression analyzed by RT-qPCR; and (**C**) number of adipocytes per area (mm^2^). Groups: adult control rats fed a normal-fat diet (NF-Control) and adult rats that were treated during lactation with vehicle (Control), leptin, celastrol, or leptin and celastrol (Lep+Cel), and subsequently pair-fed for 11 weeks with high-fat diet (HF) administered under isocaloric conditions to the NF-Control group. In (**B**), data represent threshold cycle (Ct) values. The number of animals in each group in which *Ucp1* gene expression was detected (+animals) is indicated. In (**C**), data represent means ± SEMs (n = 6–10). The # symbol shows the significance of all the HF-fed groups vs. the NF-Control group (U Mann–Whitney, *p* < 0.05); the * symbol shows the significance of the HF-treated groups vs. the HF-Control group (U Mann–Whitney, *p* < 0.05).

HF pair-feeding induced the expression of some brown-brite markers in a depot-specific manner. In rWAT, morphological and immunohistochemical analysis revealed the appearance of UCP1-positive multilocular adipocytes in animals fed an HF diet. This browning induction was mainly evident in those animals that received leptin during the suckling period and in those co-treated with leptin+celastrol (Figure 6A). For *Ucp1* gene expression analysis, we directly used the Ct values obtained in the RT-qPCR. This is because this gene has an ectopic expression in WAT, and therefore, it was not expressed in all the animals analyzed. Remarkably (Figure 6B), the Ct values obtained in the RT-qPCR were lower in the HF-Leptin animals (and in those of the HF-Cel+Lep group), confirming the results of immunohistochemistry and evidencing greater *Ucp1* mRNA expression levels in this group. Moreover, *Ucp1* mRNA was detected in 100% of the animals analyzed in the HF-Leptin group. Regarding the treatment with celastrol, animals of the HF-Celastrol group were those with fewer signs of browning induction in the rWAT. In this line, these animals presented larger adipocytes in this adipose depot, as evidenced by a lower number of adipocytes per area (Figure 6C). Transcriptomic analysis performed in eWAT did not show *Ucp1* gene expression in any of the groups studied. In addition to UCP1, other brown-brite gene expression markers were analyzed in the two WAT depots. In the rWAT (Figure 4), *Adrβ3* gene expression was increased in HF-Lep and HF-Lep+Cel compared to NF-Control animals (U Mann–Whitney, *p* < 0.05), and *Cidea* and *Pgc1α* mRNA levels were increased in Lep+Cel vs. HF-Control animals (U Mann–Whitney, *p* < 0.05). Other brown-brite markers (*Hoxc9* and *Prdm16*) were not affected by HF diet feeding or perinatal treatment in rWAT. In the eWAT (Figure 5), HF diet feeding induced the *Cidea* expression regardless of suckling treatment, and *Hoxc9* expression was only greater in the HF-Leptin group. The other brown markers studied (*Pgc1α* and *Prdm16*) were not detected in a representative number of animals (minimum of three animals per group) in the eWAT.

Regarding leptin-signaling-related genes, in rWAT (Figure 4), Lep mRNA expression increased in the HF-Lep+Cel group and *Socs3* in the HF-Celastrol and HF-Lep+Cel groups, while *Stat3* expression decreased in the HF-Leptin group (U Mann–Whitney *p* < 0.05). In eWAT (Figure 5), increased expression of *Socs3* was also observed in the HF-Celastrol and HF-Lep+Cel groups (U Mann–Whitney *p* < 0.05).

### 3.5. Brown Adipose Tissue Gene Expression Analysis

**Figure 7:**
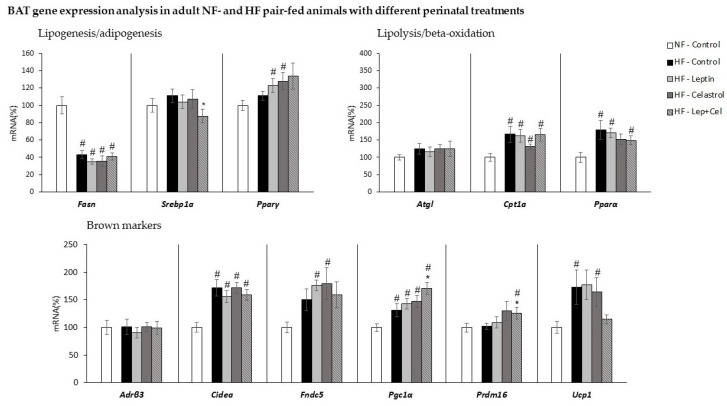
Brown adipose tissue (BAT) gene expression analysis in adult control rats fed a normal-fat diet (NF-Control) and in adult rats that were treated during lactation with vehicle (Control), leptin, celastrol, or leptin and celastrol (Lep+Cel) and subsequently pair-fed for 11 weeks with high-fat diet (HF) administered under isocaloric conditions to the NF-Control group. mRNA expression was measured by RT-qPCR. Data represent means ± SEMs (n = 8–10) of ratios of specific mRNA levels relative to *Gdi* (used reference gene), expressed as a percentage of the value of the NF-Control group that was set to 100%. Statistics: The # symbol shows the significance of all the HF-fed groups vs. the NF-Control group (U Mann–Whitney, *p* < 0.05); the * symbol shows the significance of the HF-treated groups vs. the HF-Control group (U Mann–Whitney, *p* < 0.05).

As seen in Figure 7, in BAT, and in the same way as in WAT, HF diet pair-feeding induced a compensatory adaptation consisting of decreased *Fasn* and increased *Cpt1a* expression, regardless of the suckling treatment (U Mann–Whitney *p* < 0.05). In addition, a greater mRNA expression of the lipolytic gene Pparα was observed in HF-treated animals, except in the HF-Celastrol group. Contrary to what observed in WAT, the expression of the lipolytic gene *Atgl* was not affected by dietary or suckling treatment.

Regarding markers of thermogenesis activation, increased expression of key brown marker *Ucp1* was only found in the HF groups treated with vehicle or celastrol (HF-Control and HF-Cel) (U Mann–Whitney *p* < 0.05). Other brown adipocyte markers, *Cidea* and *Pgc1α,* increased their expression in HF-fed animals, regardless of the suckling treatment, while *Fndc5* and *Prdm16* only presented greater gene expression levels in the HF-Leptin and HF-Celastrol, and in the HF-Lep+Cel group, respectively (U Mann–Whitney *p* < 0.05). Of note, mRNA expression for *Pgc1α* and *Prdm16* was greater in the HF-Lep+Cel group not only compared to NF control animals but also to HF-fed controls. The expression of *Adrβ3* was not affected by dietary and suckling treatment in BAT.

### 3.6. Hypothalamic Gene Expression Analysis

**Figure 8:**
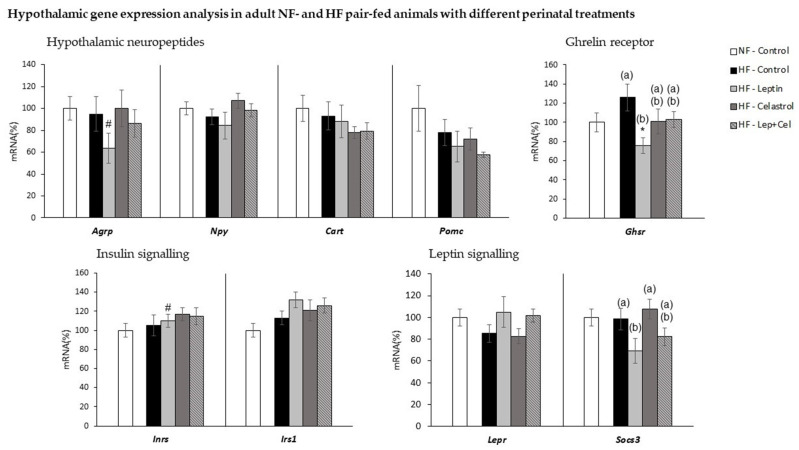
Hypothalamic gene expression analysis in adult control rats fed a normal-fat diet (NF-Control) and in adult rats who were treated during lactation with vehicle (Control), leptin, celastrol, or leptin and celastrol (Lep+Cel) and subsequently pair-fed for 11 weeks with high-fat diet (HF) administered under isocaloric conditions to the NF-Control group. mRNA expression was measured by RT-qPCR. Data represent means ± SEMs (n = 8–10) of ratios of specific mRNA levels relative to *Gdi* (used reference gene), expressed as a percentage of the value of the NF-Control group that was set to 100%. Statistics: One-way ANOVA was performed only in the HF-fed animals. Data not sharing a common letter (a, b) are significantly different. LSD post hoc was used after ANOVA analysis. The # symbol shows the significance of all the HF-fed groups vs. the NF-Control group (U Mann–Whitney, *p* < 0.05); the * symbol shows the significance of the HF-treated groups vs. the HF-Control group (U Mann–Whitney, *p* < 0.05).

As evidenced in Figure 8, no relevant changes were observed at the transcriptional level in the hypothalamus in response to the intake of an isocaloric HF diet. However, leptin treatment during lactation resulted in inhibition of hypothalamic expression of the ghrelin receptor (*Ghsr*) gene, as well as the negative regulator of the leptin signaling, *Socs3* (one-way ANOVA, *p* < 0.05), compared with HF-Control animals. In addition, compared with normal-fat-fed animals, the hypothalamic expression of the orexigenic neuropeptide *Agrp* was decreased and the expression of the insulin substrate receptor (*Irs1*) was increased in the HF-Leptin animals (U Mann–Whitney *p* < 0.05). Gene expression of the other hypothalamic neuropeptides, *Npy*, *Cart,* and *Pomc*, and of the leptin receptors, was not affected by HF diet feeding or suckling treatment.

## 4. Discussion

The prevalence of MONW features among the population is increasingly common and could be a health concern in the near future [34,35]. One of the main causes involved in the appearance of this phenotype is the intake of unbalanced diets [16,17,18,19]. This is supported by our previous studies, where long-term feeding in adult rats with an HF diet administered under isocaloric conditions to a balanced control diet (pair-feeding) resulted in increased adiposity and in adiposity-related metabolic alterations, even in the absence of increased body weight, mimicking the MOWN phenotype [36,37]. It is well known that early life nutrition has a clear influence on the metabolic phenotype in adulthood [1]. In this sense, previous studies by our group revealed that leptin treatment during the suckling period prevents diet-induced obesity later in life [7,8]. With this background, we aimed to evaluate the possible protective role of perinatal treatment with leptin and celastrol, the latter of which has been described as a leptin sensitizer with anti-obesity properties [13], and their combination, against metabolic alterations related to the diet-induced MOWN phenotype.

In the present work, we mimicked the MOWN phenotype by the administration of an HF pair-fed diet to male Wistar rats for 11 weeks after weaning. HF-fed animals presented the same body weight as NF-controls but with greater adiposity, including liver fat accumulation and alteration in serum parameters indicative of insulin resistance. However, the acquisition of the MONW phenotype and related metabolic complications was conditioned by perinatal treatment with leptin and/or celastrol. The chronic administration of physiological doses of leptin during suckling did not result in significant effects on body weight or body fat in the pre-weaning period, as we have previously reported [8,24]. However, this perinatal treatment with leptin protected against key features of the MONW phenotype in adulthood when animals were exposed to an isocaloric hyperlipidic diet after weaning. Thus, HF pair-fed animals that were treated with leptin did not present greater total fat content in the liver, and although adiposity index was not decreased in comparison to the control HF-fed animals, they showed a lower size of the epididymal WAT depot and lower levels of circulating leptin. Moreover, despite chronic intake of the HF diet, leptin-treated animals did not show signs of insulin resistance. Furthermore, transcriptomic analysis of relevant genes in key homeostatic tissues provided additional information of interest. One of the relevant places of leptin action is the hypothalamus. We have previously described that leptin administration during lactation induces transcriptional changes in the hypothalamus that contribute to protection against obesity when animals are exposed to ad libitum intake of an HF diet in adulthood [8]. In the same line, in the present study, we report transcriptomic effects of leptin treatment at the hypothalamic level that could contribute to protection against fat accumulation and adiposity-related damage associated with the MONW phenotype. Thus, leptin-treated animals presented decreased hypothalamic expression of the gene coding for the receptor of the orexigenic signal ghrelin (*Ghrs*) as well as decreased expression of the gene coding of the orexigenic agouti-related peptide (*Agrp*). They also presented greater hypothalamic expression of the gene coding for the insulin receptor substrate 1 (*Irs1*), which could be indicative of greater insulin sensitivity capacity. This is in line with the lack of insulin resistance observed in animals treated with leptin in comparison to control, non-treated MONW rats. In addition, decreased expression of the suppressor of cytokine signaling 3 gene, *Socs3*, also observed in the leptin-treated group, is suggestive of increased action of leptin on the hypothalamus. Hypothalamic leptin action induces a well-known antilipolytic and thermogenic effect on adipose tissue mediated by the beta 3-adrenergic receptor [38]. Consistently, perinatal leptin treatment increased gene expression of *Adrβ3*, of the lypolitic *Atgl* gene, and induced the appearance of brown-like adipocytes expressing UCP1, as observed by immunohistochemical and gene expression analysis in the retroperitoneal WAT. However, despite HF-Leptin animals presented a lower size of the epididymal WAT and greater levels of the brite marker *Hoxc9*, no *Ucp1* gene expression was evident, suggesting a depot-specific susceptibility to browning induction, as we have previously described [33].

Celastrol is a potent leptin-sensitizing anti-obesity agent when administered to adult animals [13]. The administration of celastrol to obese adult rodents has been reported to lead to reduced food intake, accompanied by decreased body weight and body fat, and improved glucose tolerance and insulin sensitivity [13,39,40]. Accordingly, in the present study, celastrol treatment during the suckling period resulted in reduced body weight at the end of lactation. However, unexpectedly, no protective effect was evident when these animals that had received perinatal celastrol treatment were subsequently exposed to an unbalanced isocaloric HF diet for 11 weeks after weaning. In fact, treatment resulted in even greater rWAT size, with larger adipocytes, and higher ad libitum circulating glucose levels and fasting insulin levels. Celastrol treatment in adult animals has also been reported to increase leptin sensitivity [13], ameliorate non-alcoholic fatty liver disease [14], and increase iWAT browning and BAT activation [41], which protect against diet-induced obesity. However, perinatal celastrol treatment in our animals did not protect them from MONW phenotype features. Not only that, contrary to what was expected considering the reported effect of celastrol in adult animals, we found a markedly higher expression of the negative regulator of the leptin signaling, *Socs3,* in the two WAT depots studied, rWAT and iWAT, pointing towards decreased leptin sensitivity. We hypothesize that the administration of a leptin-sensitizing compound in this critical perinatal period could lead to metabolic imprinting, causing leptin insensitivity as an adaptive protective mechanism. However, this imprinting would result in an unfavorable metabolic outcome if these animals are exposed to an obesogenic environment in adulthood. In addition, in comparison to HF-Control animals with MONW phenotype, no protective effect against liver fat deposition and no relevant changes were observed in adipose tissue browning potential or in BAT thermogenic capacity in these celastrol-treated animals.

The co-administration of leptin and celastrol to diet-induced obese adult rodents has been shown to exert a more potent anorectic and body weight-reducing effect than leptin administered alone [13]. In our study, the co-administration of leptin together with celastrol during suckling was able to counteract some of the alterations produced by celastrol treatment in adipose tissue. Therefore, leptin co-administration protected from the increase in rWAT size observed in the HF-Celastrol group and ameliorated circulating leptin levels. In fact, animals from the HF-Lep+Cel group showed increased signs of browning in rWAT, evidenced by the appearance of UCP1-positive multilocular adipocytes, which is consistent with greater *Ardβ3* mRNA levels. Accordingly, greater expression of *Ucp1* and of brown-adipocyte markers (*Cidea* and *Pgc1α*) was observed in the rWAT of the HF-Lep+Cel group. In addition, consistent with a greater thermogenic capacity, treatment with both compounds increased the size of BAT as well as the expression in this tissue of the brown adipocyte marker *Prdm16*.

Taken together, our results reinforce the perinatal programming role of leptin in preventing obesity-related disorders; in this case, preventing the appearance of an MONW phenotype, which had not been previously reported. Moreover, we provide evidence that the administration of celastrol, which has been widely demonstrated as an anti-obesity compound when administered to adult animals, does not protect from the development of MONW when administered in the perinatal period. Not only that, perinatal administration of celastrol could program a worse efficacy against an obesogenic environment in adulthood. More research is needed to clarify the possible deleterious effects of celastrol if administered during lactation, alterations that appear to be partially preventable by the co-administration of leptin.

## Figures and Tables

**Table 1:** Body weight and adiposity and serum parameters measured in adult control rats fed a normal-fat diet (NF-Control) and in adult rats that were treated during lactation with a vehicle (Control), leptin, celastrol, or leptin and celastrol (Lep+Cel), and subsequently pair-fed for 11 weeks with high-fat diet (HF) administered under isocaloric conditions to the NF-Control group.

**Body Weight and Adiposity Parameters**					
	**NF**	**HF Pair-Fed**			
	**Control**	**Control**	**Leptin**	**Celastrol**	**Lep+Cel**
Weight (g)	409 ± 13	416 ± 6	410 ± 6	420 ± 6	427 ± 5
Fat mass (%)	19.4 ± 0.5	23.9 ± 1.8 # (a)	22.9 ± 1.2 # (a)	27.3 ± 0.8 # (b)	25.6 ± 0.7 # (a,b)
Adiposity index (%)	8.36 ± 0.26	11.2 ± 0.6 # (a,b)	10.4 ± 0.4 # (a)	11.9 ± 0.3 # (b)	11.0 ± 0.3 # (a,b)
iWAT (g)	9.73 ± 0.86	13.9 ± 1.2 #	13.5 ± 0.9 #	16.2 ± 0.7 #	14.9 ± 0.8 #
eWAT (g)	9.59 ± 0.66	13.7 ± 0.5 # (a)	11.1 ± 0.5 (b) *	13.1 ± 0.7 #(a)	12.2 ± 0.6 # (a,b)
mWAT (g)	4.04 ± 0.26	5.46 ± 0.4 # (a,b)	4.83 ± 0.30 # (a)	4.91 ± 0.36 #(a)	6.04 ± 0.24 # (b)
rWAT (g)	11.0 ± 0.9	13.1 ± 0.4 (a)	13.2 ± 0.7 (a)	15.8 ± 0.8 # (b) *	13.7 ± 0.7 (a)
BAT (g)	0.65 ± 0.05	0.71 ± 0.04	0.68 ± 0.04	0.80 ± 0.09	0.85 ± 0.06 #
**Serum Parameters**					
	**NF**	**HF Pair-Fed**			
	**Control**	**Control**	**Leptin**	**Celastrol**	**Lep+Cel**
Glucose Fasting(mg/dL)	101 ± 3	120 ± 7 #	114 ± 4	113 ± 5	122 ± 4 #
Glucose Ad libitum (mg/dL)	110 ± 2	111 ± 2 (a)	116 ± 2 (a)	120 ± 3 # (a,b) *	127 ± 5 # (b) *
Insulin Fasting (µg/L)	0.218 ± 0.046	0.330 ± 0.053	0.243 ± 0.027	0.408 ± 0.073 #	0.341 ± 0.071
HOMA-IR	1.06 ± 0.16	2.45 ± 0.52 #	1.69 ± 0.24	2.69 ± 0.48 #	2.49 ± 0.54 #
Leptin (mg/mL)	12.8 ± 1.0	18.1 ± 2.3 # (a)	12.5 ± 1.3 (b) *	18.7 ± 0.5 # (a)	16.6 ± 1.3 # (a,b)

Data represent means ± SEMs (n= 8-10). Statistics: One-way ANOVA was performed for parameters measured only in the HF-fed animals. Data not sharing a common letter (a, b) are significantly different. LSD post hoc was used after ANOVA analysis. The # symbol shows the significance of all the HF-fed groups vs. the NF-Control group (U Mann–Whitney, *p* < 0.05); the * symbol shows the significance of the HF-treated groups vs. the HF-Control group (U Mann–Whitney, *p* < 0.05).

## Data Availability

Data available on request.

## Supplementary Materials

The following are available online at https://www.mdpi.com/article/10.3390/nu14112277/s1, Table S1: Nucleotide sequences of primers used for RT-qPCR amplification, as well as amplicon size.
