# Supplementary material for: Perinatal Treatment with Leptin, but Not Celastrol, Protects from Metabolically Obese, Normal-Weight Phenotype in Rats
Source: Nutrients. 2022 May 29;14(11):2277. doi: 10.3390/nu14112277 (PMC9183119; doi:10.3390/nu14112277)
Supplement: Supplementary file 1 [file nutrients-14-02277-s001.zip › nutrients-1734671-supplementary.pdf]

Table S1. Nucleotide sequences of primers used for RT-qPCR amplification, as well as amplicon size.

Gene	Forward Primer (5′-3′)	Reverse Primer (5′-3′)	Amplicon Size (bp)
Adrβ3	CAGTGGTGGCGTGTAGGG	AAGGAGACGGAGGAGGAGAG	113
Agrp	AGAGTTCTCAGGTCTAAGTCT	CTTGAAGAAGCGGCAGTAGCACGT	210
Atgl	TGTGGCCTCATTCCTCCTAC	AGCCCTGTTTGCACATCTCT	271
Cart	AGAAGAAGTACGGCCAAGTCC	CACACAGCTTCCCGATCC	84
Cidea	TCAGACCCTAAGAGACAACACA	CATTGAGACAGCCGAGGA	164
Cpt1a	CGAGAAGGGAGGACAGAGAC	GGACACCACATAGAGGCAGAA	201
Cpt1β	GCAAACTGGACCGAGAAGAG	CCTTGAAGAAGCGACCTTTG	180
Fasn	CGGCGAGTCTATGCCACTAT	ACACAGGGACCGAGTAAT	222
Fndc5	ACCTGGAGGAGGACACAGAA	CCACATGAAGAGGACCACAA	215
Ghsr	TCAGCCAGTACTGCAACCTG	GGAGAGATGGGATGTGCTGT	222
Hoxc9	CGGCAGCAAGCACAAAGA	AGAAACTCCTTCTCCAGTTCCA	138
Hsp90	AGTTCGATGGCAAGAGCCTG	GGGGGAAGACACAAGCCTAT	185
Insr	CTCCTGGGATTCATGCTGTT	GTCCGGCGTTCATCAGAG	242
Irs1	GCAACCGCAAAGGAAATG	ACCACCGCTCTCAACAGG	293
Lep	TTCACACACGCAGTCGGTAT	AGGTCTCGCAGGTTCTCCAG	186
Lepr	AGCCAAACAAAAGCACCATT	TCCTGAGCCATCCAGTCTCT	174
Npy	TGGACTGACCCTCGCTCTAT	GTGTCTCAGGGCTGGATCTC	188
Pgc1a	CATTTGATGCACTGACAGATGGA	CCGTCAGGCATGGAGGAA	70
Pomc	CCTGTGAAGGTGTACCCCAATGTC	CACGTTCTTGATGATGGCGTTC	266
Ppara	TGTCGAATATGTGGGGACAA	AAACGGATTGCATTGTGTGA	215
Pparγ	AGACCACTCGCATTCCTTTG	TCGCACTTTGGTATTCTTGG	154
Prdm16	ACGACCACCTCTGCTACCTC	GGGCTCCTATTTTTGACCTTCT	216
Srebp1a	CCCACCCCCTTACACACC	GCCTGCGGTCTTCATTGT	198
Socs3	ACTGAGCCGACCTCTCTCCT	CCCCTCTGACCCTTTCTTTG	172
Stat3	GCTGACCAATAACCCCAAGA	ACACCCTGAGTAGTTCACACCA	181
Ucp1	GGGCTGATTCCTTTTGGTCT	GGTGGTGATGGTCCCTAAGA	229
Gdi1 (Reference gene)	CCGCACAAGGCAAATACATC	GACTCTCTGAACCGTCATCAA	159
